# VLA-1 Binding to Collagen IV Controls Effector T Cell Suppression by Myeloid-Derived Suppressor Cells in the Splenic Red Pulp

**DOI:** 10.3389/fimmu.2020.616531

**Published:** 2021-01-18

**Authors:** Ina N. Eckert, Eliana Ribechini, Katja J. Jarick, Sandra Strozniak, Sarah J. Potter, Andreas Beilhack, Manfred B. Lutz

**Affiliations:** ^1^ Institute for Virology and Immunobiology, University of Würzburg, Würzburg, Germany; ^2^ Department of Internal Medicine II, University Hospital Würzburg, Würzburg, Germany

**Keywords:** myeloid-derived suppressor cells (MDSCs), T cells, VLA-1, homing, spleen

## Abstract

Myeloid-derived suppressor cells (MDSCs) represent a major population controlling T cell immune responses. However, little is known about their molecular requirements for homing and T cell interaction to mediate suppression. Here, we investigated the functional role of the homing and collagen IV receptor VLA-1 (α1β1-integrin) on *in vitro* GM-CSF generated murine MDSCs from wild-type (WT) and CD49a/α1-integrin (*Itga1* ^−/−^) gene-deficient mice. Here, we found that effector (Teff) but not naive (Tn) CD4^+^ T cells express VLA-1 and monocytes further up-regulated their expression after culture in GM-CSF when they differentiated into the monocytic subset of resting MDSCs (R-MDSCs). Subsequent activation of R-MDSCs by LPS+IFN-γ (A-MDSCs) showed increased *in vitro* suppressor potential, which was independent of VLA-1. Surprisingly, VLA-1 deficiency did not influence A-MDSC motility or migration on collagen IV *in vitro*. However, interaction times of *Itga1* ^−/−^ A-MDSCs with Teff were shorter than with WT A-MDSCs on collagen IV but not on fibronectin substrate *in vitro*. After injection, A-MDSCs homed to the splenic red pulp where they co-localized with Teff and showed immediate suppression already after 6 h as shown by inhibition of T cell proliferation and induction of apoptosis. Injection of A-MDSCs from *Itga*1^−/−^ mice showed equivalent homing into the spleen but a reduced suppressive effect. Interaction studies of A-MDSCs with Teff in the subcapsular red pulp with intravital two-photon microscopy revealed also here that MDSC motility and migration parameters were not altered by VLA-1 deficiency, but the interaction times with Teff were reduced. Together, our data point to a new role of VLA-1 adhesion to collagen IV as a prerequisite for extended contact times with Teff required for suppression.

## Introduction

MDSCs were initially identified in tumor-bearing mice and other diseases such as infections, trauma and chronic inflammation (1, 2). MDSCs can control T cell immune responses and their major role in modulating T cell responses against tumors is well established. Murine MDSCs can be subdivided into granulocytic (CD11b^+^ Ly-6C^low^ Ly-6G^+^) and monocytic (CD11b^+^ Ly-6C^high^ Ly-6G^-^) subsets (3–5). However, this marker profile is insufficient to distinguish non-suppressive monocytes or granulocytes from their suppressive MDSC counterparts, respectively, or to dissect differentiated MDSCs from their immediate precursor stages (1). We established a protocol to generate murine MDSCs from bone marrow (BM) (6). This protocol allowed us to study the stepwise signaling events for MDSC generation. We found that monocytes may not directly convert into A-MDSCs *in vitro* but may require GM-CSF signaling as an intermediate step of “monocyte licensing,” a state that could be also considered as “resting monocytic MDSCs” (R-MDSCs). *In vivo*, repetitive injections of GM-CSF into mice led to R-MDSC accumulation in the spleen and microbial stimulation converted them into monocytic A-MDSCs, which mediated iNOS-dependent suppression (7). Although the spleen has been identified as a critical organ to coordinate MDSC functions (5, 8), MDSC homing into the spleen and their migration within the spleen remains largely unclear. In mycobacteria-vaccinated mice MDSC accumulation occurred preferentially in the red pulp and bridging channels. An inflammatory challenge directed them into the white pulp and activated them to induce dendritic cells (DC) killing but left the white pulp T cells unaffected (9).

Lymph node and splenic white pulp homing of T cells is a typical feature of naive T cells (Tn), while Teff step-wise lose this property and acquire a different homing pattern. Specific homing receptors that guide Teff include the “very late antigen” (VLA) integrin family members that are composed of different α1-α6 chains pairing with the β1 integrin chain (10). The expression of the integrin α1β1 (VLA-1, CD49a/CD29) appears 5–6 days after activation at the T cell surface and is kept as a typical memory marker. VLA-1 is functioning mainly as collagen IV receptor and poorly binds other extracellular matrix (ECM) components (11–14). The red pulp is the only anatomical region in the spleen where collagen IV is freely accessible for interaction with cells (15, 16) leading to the accumulation of Teff in the red pulp (17–20). VLA-1^+^ Teff are generated at frequencies of 8–10% among splenic Teff and up to 30% at infection sites such as the lung (21). Thus, Tn cell priming occurs in the paracortex of lymph nodes or the white pulp of the spleen (T cell areas) but VLA-1^+^ Teff are detected outside these areas, mainly in the red pulp.

A major function of MDSCs is to control immune responses during late stages of infections or during chronic inflammation to limit immunopathology, rather than to prevent the initial priming of immune responses against pathogens (22). In this scenario, MDSCs should accumulate and act preferentially on Teff, rather than on naive T cells. Since T cell priming occurs in the lymph nodes or the splenic white pulp, Teff accumulation and control can be detected rather in the red pulp of the spleen (8). In fact, this has been observed for MDSC localization in tumor-bearing mice (20, 23). However, MDSCs can express also the lymph node homing receptors CD62L (6) and CCR7 (24), enabling their homing also to sites of T cell priming. In addition, both Ly-6C^high^ monocytes and Ly-6C^high^ monocytic MDSCs express CCR2 to home to their target organs such as inflammatory sites or tumors (25, 26). Thus, MDSCs may have different options for homing, depending on the type of inflammation, infection or tumor disease and the early or late stage of an immune response. Here we investigated VLA-1 expressed by monocytic MDSCs as a homing receptor and interaction partner for collagen IV in the spleen. Surprisingly, VLA-1 did not guide MDSC homing into the spleen or influence MDSC migration behavior on collagen IV, but instead collagen IV interaction was required by MDSCs to interact with Teff as a meeting platform.

## Materials and Methods

### Mice and Animal Experiments

C57BL/6 were originally purchased from Charles River. C57BL/6, C57BL/6 albino, OT-II.dsRed, OT-II.CD90.1, and *Itga1* ^−/−^ mice (27) were bred in the animal facilities of the Institute of Virology and Immunobiology at the University of Würzburg under specific pathogen-free conditions. All animal experiments were performed after permission of and under control of the local government (Regierung von Unterfranken, AZ 55.2-2532-2-200).

### MDSC Generation and Activation

MDSCs were generated from murine BM as described in detail before (6). Briefly, BM single cell suspensions were cultured for 3 days with murine recombinant GM-CSF (200 U/ml, Peprotech) or the equivalent amount of supernatant from a GM-CSF producing cell line (28). To obtain activated monocytic MDSCs (A-MDSCs) releasing NO as a suppressor molecule, LPS (100 ng/ml, Sigma Aldrich) and IFN-γ (100 U/ml, Peprotech) were added as described (5) for 4 h or overnight as indicated.

### FACS Analyses

To block FcγR II/III interactions cells were incubated with 10% hybridoma clone 2.4G2 supernatant (ATCC) in the dark for 20 min on ice. Cell surface markers were stained in PBS containing 0.1% BSA (Serva), 0.1% sodium azide (Roth) and 10% hybridoma clone 2.4G2 supernatant in the dark for 15 to 30 min on ice. For staining intranuclear antigens, the cells were incubated with Cytofix/Cytoperm solution (eBiosciences) for 30 min at room temperature and subsequent antibody staining was performed with Perm buffer (BD Pharmingen) for 45 to 60 min at room temperature. For annexin V staining, the cells were stained with 0.5 μl annexin V in 50 μl annexin V binding buffer. After 15 min incubation at room temperature, 100 μl annexin V binding buffer were added and the samples were acquired within 1 h. All samples were measured using the BD LSR II Flow Cytometer and the data was analyzed *via* FlowJo 10 (Tree Star) and Prism 5 or 7 (GraphPad). Anti-mouse directly conjugated antibodies CD11b-PerCP-Cy5.5 (M1/70), CD11b-Alexa Fluor 700 (M1/70), Ly6G-PerCP-Cy5.5 (1A8), Ly6G-APC/Fire (1A8), Ly6C-Alexa Fluor 647 (HK1.4), Ly6C-Brilliant Violet 510 (HK1.4), CD49a-PE (HMα1), CD49a-APC (HMα1), Thy1.1-PerCP-Cy5.5 (30-H12), CD4-PerCP-Cy5.5 (GK1.5), CD8-APC (53-6.7), CD25-PE-Cy7 (PC61), CD62L-Alexa Fluor 700 (Mel14), CD69-Alexa Fluor 488 (H1.2F3), CD69-APC (H1.2F3), Ki-67-Alexa Fluor 647 (16A8), Ki-67-FITC (16A8), iNOS-FITC or -PE (XCNFT), and annexin V-FITC were all purchased from Biolegend.

### Tn and Teff Preparation and T Cell Suppressor Assays

Lymph nodes and spleens were collected from C57BL/6, OT-II.dsRed or congenic OT-II.CD90.1 mice and processed until cell suspension following standard protocols. Upon erythrocyte lysis, cells were cultured for 6 days in a 24-well plate seeded at a concentration of 2 × 10^6^ cells/well in the presence of 1 µM OVA_323-339_ peptide. At days 6–7, cells were collected and tested by FACS for their surface expression of VLA-1 and effector markers. As counterpart, Tn freshly isolated from the same organs of littermate mice were used in parallel for *in vitro* suppression assays. Teff were kept in culture and re-stimulated weekly in the presence of cognate peptide and Th1 polarizing conditions with LPS-matured DCs in order to favor and maintain VLA-1 expression (29). Tn and Teff OT-II cells were used in an inhibition T cell proliferation assay by plating 20.000–200.000 T cells/well stimulated with 1 µM OVA peptide and titrations of MDSCs in a 96-well plate, round-bottomed; triplicate cultures were pooled for analyses. When syngeneic naive T cells from C57BL/6 WT mice were used, the same procedure was applied but bulk T cells were stimulated by adding anti-CD3 and anti-CD28 antibodies (2.5 µg/ml each). After 4–5 days proliferation of T cells labelled with CFSE (Sigma), CellTrace Violet (Invitrogen) or eFluor670 (Invitrogen) was measured by by flow cytometry (30). In some cases, proliferation was assessed by staining for Ki67 (2.5 µg/ml) after 3 days (31).

### Bioluminescence

For *in vivo* bioluminescence imaging (32, 33), mice were anesthetized with an intraperitoneal injection of 80 mg/kg body weight ketamine hydrochloride (Pfizer) and 16 mg/kg body weight xylazine (cpPharma). Together with anesthetics, mice were injected with 300 mg/kg body weight D-luciferin (Biosynth). Ten minutes later, bioluminescence signals of the anesthetized mice were recorded using an IVIS Spectrum imaging system (Perkin-Elmer/Caliper Life Sciences). Pictures were taken from the lateral view in automatic mode with a maximum exposure time of five min per picture. For *ex vivo* imaging, mice were injected with D-luciferin and euthanized 10 min later. Internal organs were removed and subjected to *ex vivo* bioluminescence imaging. Pictures were evaluated using Living Image 4.0 software (Caliper Life Sciences).

### MDSC Migration and Interaction With T Cells *In Vitro*

WT or *Itga1* ^−/−^ MDSCs were harvested, spun down and resuspended in complete media at a concentration of 3 × 10^6^ cells/ml into a 50-ml falcon and pre-activated by a 4-h incubation time with 1 µg/ml LPS + 0.5 µg/ml IFN-γ at 37°C. Cells were washed with PBS and CFSE-labeled. Teff OT-II cells were derived from OT-II.dsRed mice and generated by repeated stimulations with the cognate peptide under Th1 conditions. Cells were mixed at a ratio 1:2 (MDSC:T) and transferred into a μ-slide 8 well chamber (IBIDI, #80821) pre-coated with fibronectin (20 μg/ml) or collagen IV (100 μg/ml), both Sigma. Cell-interactions were imaged using an inverted Confocal Laser Scanning Microscope (Zeiss LSM 780) with an XL incubator special for live cell imaging. Acquisition was performed using a 10× objective and acquiring consecutive pictures in 4 different quadrants every 15 s for a total of 60 min (approx. 200 cycles).

### Fluorescence Microscopy

Spleens from euthanized mice were collected and transferred into tubes containing OCT compound (Tissue-Tek, SAKURA), immediately frozen, and stored at (-80°C). Organs were cut into 10-μm-thick slices using a Cryotome (Leica), the sections were fixed for 7 min in acetone at room temperature and in case of biotinylated antibody usage subsequently treated with the Avidin/Biotin Blocking Kit (Vector Laboratories). The primary antibodies VLA-1-PE (clone HMa1), CD90.1-Biotin (OX-7), CD169-Alexa Fluor 647 (3D6.112), CD11b-Biotin (M1/70) and CD11b-Alexa Fluor 488 (M1/70) (all Biolegend) were diluted 1:100 in PBS with 2% FCS and incubated for 1 h at room temperature in a wet chamber. Slides were washed several times in PBS and Streptavidin-Cy3 (Biolegend) diluted 1:300 in PBS with 2% FCS was added for 30 min at room temperature. Finally, slides were repeatedly washed in PBS, dried, and mounted with Fluoromount-G (SouthernBiotech). Tissue sections were all visualized and further analyzed with a confocal fluorescence microscope (Zeiss LSM780) and the ZEN Black 8.1 software (Zeiss) and processed *via* the ImageJ 1.51h software.

### MDSC Migration *In Vivo* and Interaction With T Cells

For the *in vivo* characterization, cells were prepared as described before, mixed at a ratio 1:1 (7-10 × 10^6^ CFSE-labeled A-MDSC + 7 to 10 × 10^6^ OT-II.dsRed T cells) and transferred intravenously. 1 h later mice were anesthetized and a small incision in the left side was performed in order to expose the spleen. Cling foil was used to avoid lint sources in the microscope. The mouse was positioned on a heating pad and the exposed spleen was positioned under a glass cover slip using two custom-made holders. The tissue was kept moist using sterile 0.9% NaCl solution. A multiphoton microscope TrimScope II equipped with a titanium sapphire laser (Chameleon Ultra II, Coherent), beam splitters at 500, 570, and 655 nm, bandpass filters 420/50, 535/50, 605/70, and the photomultipliers from Lavision Biotec were used. Fluorophores were excited at a wavelength of 840 nm. The light intensity was increased as the square of penetration depth along the *z*-axis, between 5 and 30%. Images were acquired every 30 s in a field of view of 500 µm × 500 µm with a resolution of 512 × 512 pixels and in a subcapsular region with a range of 70–90 μm in the z-plane. MDSC and T cell migration and interaction were recorded for 30–90 min. Imaris (Bitplane, Zurich, Switzerland) was used for four-dimensional image analysis (x,y,z,t). The cell tracking was manually corrected and although no filters were applied to process the data, only tracks with durations >60 s were included in the analysis.

### Adoptive Co-transfer of MDSCs and T Cells for Suppression *In Vivo*

Teff were generated from C57BL/6 OT-II.CD90.1 mice. R-MDSC and A-MDSC of C57BL/6 and *Itga1* ^−/−^ WT mice were labeled with CFSE, CellTrace Violet or eFluor 670 (eBiosciences) following the manufacturer’s instructions. Briefly, the cells were centrifuged and resuspended with 2.5 µM CFSE or 1.75 µM eFluor 670 in 1 ml PBS per 2 × 10^7^ cells and incubated in the dark for 10 min at room temperature. Subsequently, the cells were washed with 1 ml FCS and 40 ml PBS. CellTrace Violet staining was performed by incubating 1 × 10^7^ cells in 1 ml PBS containing 5 μM CellTrace Violet for 6 min in the dark at room temperature and subsequent washing with 500 μl FCS and 5 ml RPMI for 5 min at 37°C. The T cells and MDSCs were injected into the lateral tail vein of C57BL/6 WT mice and the spleens were harvested at different time points. For FACS analysis, the spleens were digested with 1 mg/ml Collagenase IV (Worthington), 20 μg/ml DNase I (Roche) an 2% FCS (Gibco) for 45 min at 37°C.

### Statistics

Comparisons of data were analyzed by the tests indicated in each figure legend for the various types of assays using GraphPad Prism 5.0. In some cases, the Student’s t test with EXCEL 14.5.3 was used. Data from the experiments are presented as mean values ± SEM or SD, as indicated. Differences of p < 0.05 were considered significant.

## Results

### VLA-1 Expression, Adhesion Properties, and Functional Role for *In Vitro* Suppression

**Figure 1:**
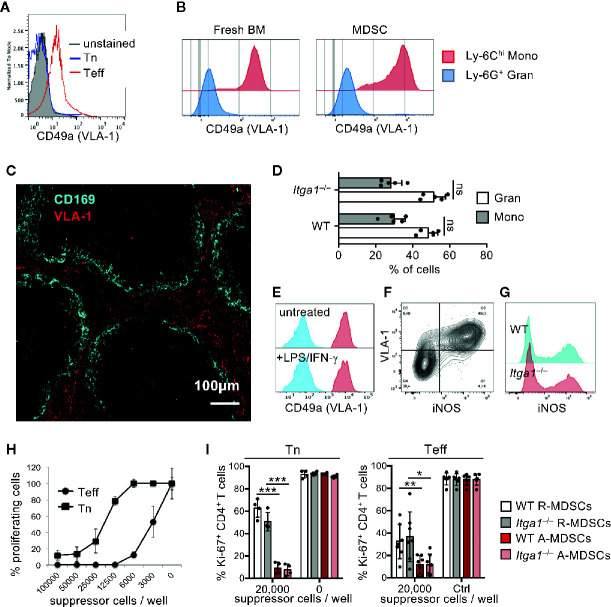
VLA-1 expression marks Teff, monocytic MDSCs and the splenic red pulp but does not influence suppression *in vitro*. **(A)** OT-II cells were stained freshly (Tn) or after 1 week of stimulation (Teff) with α1-integrin antibody to detect VLA-1 expression by FACS. Representative of n=3 experiments. **(B)** Fresh BM cells were directly analyzed or cultured for 3d with GM-CSF and stained with with α1-integrin antibody to detect VLA-1 expression on CD11b^+^ Ly-6G^-^ Ly-6C^hi^ subsets of monocytic or CD11b^+^ Ly-6G^+^ Ly-6C^low^ granulocytic MDSC subsets by FACS of WT and *Itga1* ^−/−^ cells. Representative of n>10 experiments. **(C)** WT spleen sections were stained for VLA-1 and CD169 to determine the red and white pulp areas. Representative of n=3 experiments. **(D)** BM cells of WT mice were cultured for 3d with GM-CSF and FACS-stained. Frequencies of CD11b^+^ Ly-6G^+^ and CD11b^+^ Ly-6C^+^ cells displayed. Mean ± sd of n=5 experiments. **(E)** BM cells of WT and *Itga1* ^−/−^ mice were cultured for 3d with GM-CSF and stimulated with LPS+IFN-γ overnight. CD49a expression (red) on CD11b^+^ Ly-6G^-^ Ly-6C^hi^ gated cells is shown over unstained cells (blue). Representative of n=6 experiments. **(F)** BM cells of WT mice were cultured for 3d with GM-CSF and stimulated with LPS+IFN-γ overnight. FACS analysis of the MDSC was performed for surface VLA-1 and intracellular iNOS expression. Cells gated as CD11b^+^ Ly6G^-^ Ly6C^hi^ are shown. Representative for n = 3 experiments. **(G)** Day 3 cultures of CD11b^+^ Ly6G^-^ Ly6C^hi^ MDSC of WT and *Itga1* ^−/−^ mice were stained for intracellular iNOS. Representative for n=3 experiments. **(H)** T cell suppressor assay using syngeneic CD3/CD28-stimulated Tn or Teff with titrated numbers of WT R-MDSCs. Representative of n=3 experiments. **(I)** T cell suppressor assay of bulk WT or *Itga1* ^−/−^ R- or A-MDSCs titrated into syngeneic CD3/CD28-stimulated Tn or Teff cell cultures (here only 2 × 10^4^ cells/well). After 3d FACS analysis was performed to measure T cell proliferation by their Ki-67 Expression by CD4^+^ cells. Pooled data of n=4 (Tn) or n=7 (Teff) independent experiments. Statistics by Student’s unpaired *t*-test, homoscedastic disturbances assumed. *p < 0.05, **p < 0.01, ***p < 0.005.

To investigate VLA-1 expression of CD4^+^ T cells, we stimulated OT-II lymph node cells for 1 week with the cognate OVA peptide antigen to generate a Teff cell phenotype or used freshly isolated Tn OT-II cells for surface CD49a staining. Our analyses confirm that only Teff but not Tn cells express VLA-1 on the cell surface (**Figure 1A**).

Monocytes have been shown to express VLA-1 as a homing receptor (14). Here we confirm that freshly isolated Ly-6C^hi^ monocytes from BM but not Ly-6G^+^ granulocytes express VLA-1 as detected by CD49a staining (**Figure 1B**). If we postulate that MDSCs have to meet VLA-1^+^ Teff for suppression, they should also express the same homing receptors. While VLA-4 expression has been described on the monocytic MDSCs (34), the expression and function of VLA-1 on MDSCs is unknown. Our data indicate that *in vitro* generated Ly-6C^hi^ monocytic R-MDSCs express VLA-1 and even at higher levels as compared with fresh Ly-6C^hi^ monocytes, while granulocytic Ly-6G^+^ cells in the cultures did not (**Figure 1B**). Since we wanted to study VLA-1 dependent homing into the spleen we tested for endogenous cell expression of VLA-1 in the spleen. We analyzed cryosections by immunofluorescence and found a strong and almost exclusive VLA-1 staining in the splenic red pulp of mice (**Figure 1C**). Together, these data indicate that both Teff and Ly-6C^hi^ monocytic R-MDSCs express VLA-1, and VLA-1 expression in the spleen is restricted to the red pulp.

VLA-1 deficiency may cause altered phenotypes of MDSCs during their generation in our cultures. Therefore, we compared the composition and functions of monocytic and granulocytic cells within both WT or *Itga1* ^−/−^ cultures. However, no differences were found for the cell subset frequencies (**Figure 1D**). LPS/IFN-γ represents a strong inducer of MDSC activation (A-MDSCs) leading to the release of NO as a major suppression mechanism for T cells (5) and dendritic cells (9). A-MDSCs showed no difference in their VLA-1 expression as compared to R-MDSCs. CD11b^+^ Ly-6G^-^ Ly6C^hi^ cells are shown (**Figure 1E**), and only CD11b^+^ Ly-6G^-^ Ly6C^hi^ VLA-1^+^ monocytic cells in MDSC cultures are induced to express iNOS after LPS/IFN-γ stimulation (**Figure 1F**). No difference for intracellular iNOS induction was detectable between WT or *Itga1* ^−/−^ cells (**Figure 1G**), postulating similar suppressor capacities.

We have shown before that IFN-γ produced by the T cells within an *in vitro* suppressor assay substantially contributed to MDSC activation and suppression capacity (5). Thus, pre-activated cells such as our Teff here are predicted to be more sensitive for MDSC-mediated suppression. The result indicates that much less R-MDSCs per well are needed for suppression of Teff cell proliferation as compared with Tn (**Figure 1H**). Since MDSC pre-activation improves their suppressor activity, we compared R- or A-MDSCs with or without genetic deficiency for VLA-1 for differences in their potential to suppress T cells. As expected suppression was stronger by A-MDSCs as compared with R-MDSCs, but no difference could be observed between WT and VLA-1 deficient MDSCs in their suppressor potential (**Figure 1I**). These data indicate that Teff can be suppressed better than Tn, A-MDSCs are better suppressors than R-MDSCs and genetic deficiency of VLA-1 does not influence their suppressor function *in vitro*.

### Homing of Injected R-MDSCs and A-MDSCs Into the Spleen

**Figure 2:**
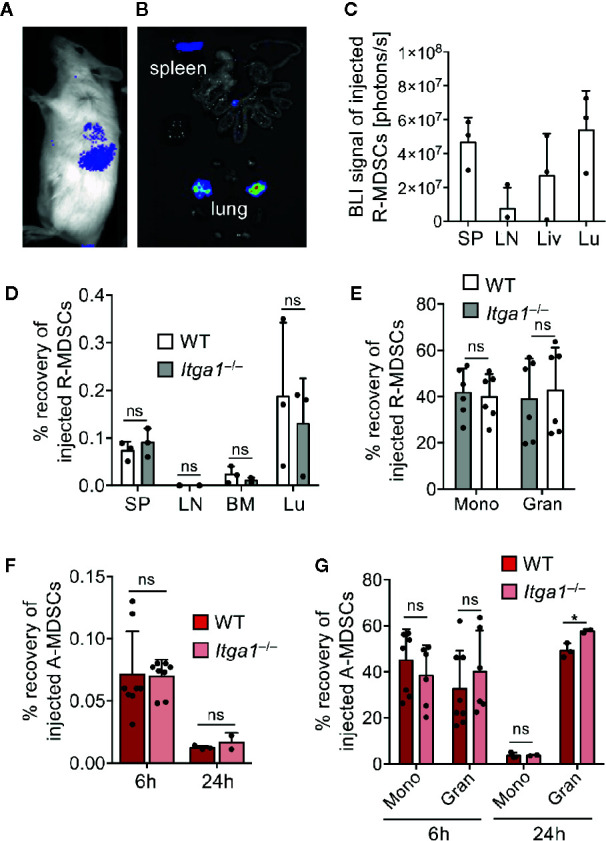
MDSC migration *in vivo*. R-MDSCs were generated from BM of Luciferease-transgeneic mice (C57BL/6) and injected i.v. into C57BL/6 albino mice. **(A)** After 24 h whole mice were imaged or **(B)** their excised organs, indicating that the spleen and the lung show a visible BLI signal. **(C)** Luciferase intensity can be visualized by spectral color display and was quantified for the indicated organs (SP, spleen; LN, lymph node; Liv, liver; Lu, lung); n=3 mice per group. **(D)** R-MDSCs derived from WT and *Itga1* ^−/−^ mice were injected i.v. into C57BL/6 WT mice (n=3) and after 6h the recovered frequency of injected cells from the indicated organs was examined *via* FACS analysis. **(E)** Subset distribution of monocytic and granulocytic cells from WT (n=6) and *Itga1* ^−/−^ (n=6) MDSC after homing to the spleen 6h after MDSC injection. **(F)** MDSCs were generated and activated for 4h with LPS+IFN-γ (A-MDSCs) and labeled with eFluor670. These A-MDSCs were injected i.v. and spleens were harvested 6 and 24 h later. Injected MDSCs were identified by CD11b and eFluor670 fluorescence. Monocytic and granulocytic subsets were further distinguished by Ly-6C and Ly-6G staining for further analyses. The frequency of recovered WT and *Itga1* ^−/−^ A-MDSCs was examined *via* FACS analysis after 6 and 24 h. **(G)** The recovered frequencies were further distinguished by their subset composition of monocytic and granulocytic cells. F+G n=2–8 mice. Statistics by unpaired Student’s t-test, homoscedastic disturbances assumed. ns, not significant, *p < 0.05.

VLA-1 expression by MDSCs is expected to be required for their adhesion to collagen IV and therefore for their homing into splenic red pulp after injection. To follow the general *in vivo* distribution after injection, R-MDSCs we prepared them from Luciferase-transgenic mice (32, 33) before intravenous administration into C57BL/6 albino mice. After 24 h luciferin was injected, and the bioluminescence was monitored of whole mice (**Figure 2A**) or the excised organs (**Figure 2B**). The quantification indicated that besides the liver and lung where small capillaries trap larger particles or cells, the preferred lymphoid organ for their homing was the spleen but not lymph nodes (**Figure 2C**). Injection of CFSE-labeled R-MDSCs from WT and *Itga1* ^−/−^ mice indicated no difference in their homing potential to lung or spleen and confirmed a lack of injected MDSCs in the lymph nodes and bone marrow (**Figure 2D**). Both granulocytic and monocytic cell subsets of WT and *Itga1* ^−/−^ mice reached the spleen (**Figure 2E**). LPS/IFN-γ activation of MDSCs has been proven to immediately initiate their suppressor function such as NO release (5, 7). To further test whether activation would change MDSC spleen homing and persistence we repeated the FACS analyses after A-MDSCs injection. After 6 h both subsets of A-MDSCs were readily detectable in the spleen but they declined strongly after 24 h, independent of their VLA-1 expression (**Figure 2F**), and monocytic A-MDSCs were lost more rapidly than the granulocytic subset (**Figure 2G**). Homing into the spleen of R-MDSCs and A-MDSCs was similar (**Figures 2D, F**). Thus, VLA-1 expression of monocytic A-MDSC did not influence their homing into the spleen. It remained to be determined whether VLA-1 expression by A-MDSCs would influence their T cell suppressor potential *in vivo*.

### WT and VLA-1 Deficient A-MDSCs Appear in the Red Pulp to Mediate Teff Suppression

**Figure 3:**
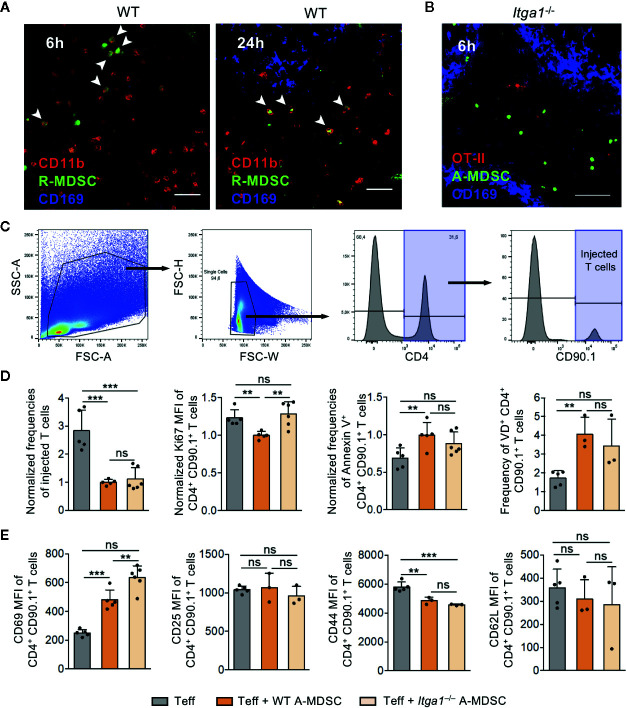
A-MDSC suppression of Teff in the red pulp is dependent on VLA-1. **(A)** CFSE-labeled WT R-MDSCs were injected i.v. and the spleens were harvested after 6 h and 24 h later for confocal microscopy of cryosections. CD11b staining marks the red pulp and CD169 staining the marginal zone. The CD11b^+^ CFSE^+^ cells, indicated by the arrows, were exclusively detected in the red pulp. Scale bar 30 µm. Representative of sections from n=3 mice. **(B)** A-MDSCs of *Itga1* ^−/−^ mice were labeled with the fluorescence marker CTV and were injected at a 1:1 ratio together with Teff of OT-II congenic CD90.1^+^ mice. Both injected cell types accumulated in the red pulp after 6 h. Scale bar 60 µm. Representative of sections from n=3-6 mice. **(C)** Experimental setup as in B, but A-MDSCs of WT or *Itga1* ^−/−^ mice were injected together with effector OT-II cells (Teff) or as a control, effector OT-II cells were injected without MDSCs. OT-II cells were analyzed by FACS for their frequency of recovery and to indicate the gating strategy. **(D)** Experimental setup and gating as in C to measure proliferation (Ki-67), or apoptosis (Annexin V or “Fixable Viability Dye,” VD). **(E)** Experimental setup as in C, but OT-II cells were analyzed for activated (CD69, CD25) or effector (CD44, CD62L) cell frequencies. n = 3–6 mice per group. Statistics by unpaired Student’s *t*-test, homoscedastic disturbances assumed. ns, not significant, ** p = 0.01, *** p = 0.005.

The spleen has been reported to represent a major organ for T cell suppression by MDSCs but the anatomical sub-localization of suppression in the spleen is not known (8, 20). During steady state monocytes form clusters in the collagen-rich subcapsular red pulp representing a reservoir and the major site for monocyte recruitment after inflammation (35). We have shown before that DC killing but not T cell suppression can be induced after infiltration of endogenous A-MDSC into the T cell areas of the white pulp (9). Whether MDSC suppression of Teff can occur in the red pulp is unclear. Although our findings so far do not argue for VLA-1 as a spleen homing receptor, we further investigated R- and A-MDSC localization after injection within different areas of the spleen. The localization of both injected CD11b^+^ WT and *Itga1*-deficient R-MDSCs could be observed at 6 and 24 h exclusively in the red pulp areas (**Figures 3A, B**). Co-injected A-MDSC and Teff were also detectable only in the red pulp (**Figure 3B**), indicating that if A-MDSC interactions occur with Teff, they have to occur in the red pulp area and that VLA-1 deficiency does not influence MDSC homing.

To test for T cell suppression, further FACS analyses of the transferred Teff was performed. Co-injection of WT A-MDSCs dramatically reduced the frequency of Teff that could be recovered (**Figures 3C, D**). The frequency of proliferating cells among the remaining T cells as detected by the Ki-67 marker was only moderately but significantly reduced and reversely, Annexin V-detected apoptosis and cell death measured by membrane permeability (‘viability dye’) were increased, indicative for immediate WT A-MDSC suppressor and killing activity already 6 h after injection (**Figure 3D**). The activation marker CD69 was increased with WT A-MDSC injection but not CD25, and the effector marker CD44 dropped while CD62L expression remained unaltered at low levels (**Figure 3E**).

Minor but significant differences between injections of WT and *Itga1* ^−/−^ A-MDSC on T cells were found only for some of the parameters tested. The Ki-67 measured proliferation rate was reconstituted as compared with WT-A-MDSC injection and CD69 expression was even more elevated (**Figures 3D, E**). Together, these data indicate that 6 h after co-injection of WT or *Itga1* ^−/−^ A-MDSCs the Teff frequency was strongly and very rapidly reduced. Among the remaining Teff moderate effects on proliferation and cell death could be detected, while the specific inhibitory measures were only partially dependent on VLA-1 expression by the A-MDSCs. However, the speed with which A-MDSCs were able to exert some significant inhibitory and even killing effects on T cells within a time window of only 6 h after injection was highly remarkable.

### A-MDSCs Deficient for VLA-1 Show a Selective Deficit for Interaction With Teff on Collagen IV *In Vitro*

**Figure 4:**
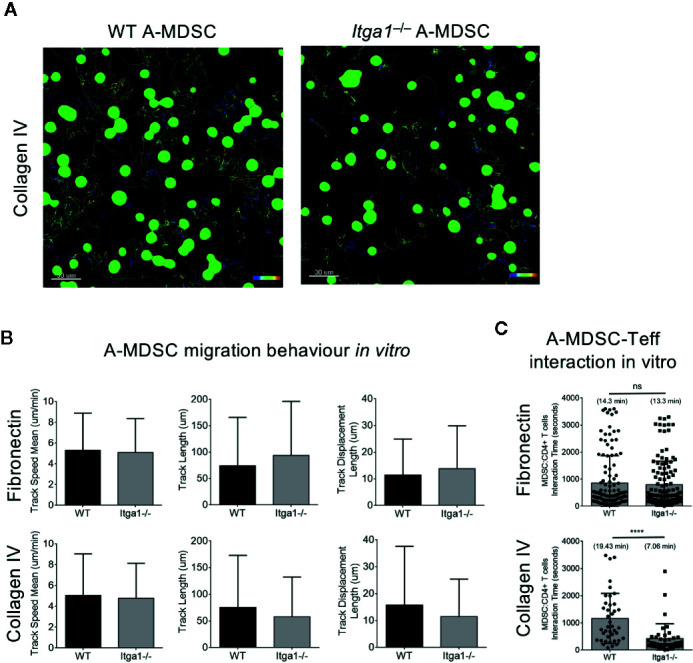
A-MDSC require VLA-1 interaction with collagen IV substrate to contact Teff but not for for motility and migration. WT or *Itga1* ^−/−^ A-MDSCs activated with LPS/IFN-γ and CFSE-labeled were mixed with Teff from OT-II.dsRed mice at a ratio 1:2 (MDSC:T) and transferred into a μ-slide 8 well chamber pre-coated with fibronectin or collagen IV. **(A)** Example of A-MDSC tracking. **(B)** Quantification of MDSC migration parameters were calculated after 60 min imaging using an inverted Confocal Laser Scanning Microscope and acquiring consecutive pictures in 4 different quadrants every 15 s. **(C)** In the same setting as in A, MDSC-T cell interactions were followed and quantified. Tracks of WT: n = 125; KO: n = 40. Numbers between brackets indicate MDSC:T cells interaction time in minutes. Statistics by unpaired Student’s *t*-test, homoscedastic disturbances assumed. ns, not significant, **** p = 0.001.

Since the NO production and suppression capacity of WT and *Itga1* ^−/−^ A-MDSCs was similar on plastic an intrinsic suppression defect by VLA-1 deficiency seems to be excluded. Therefore, the differences observed in suppression by WT and *Itga1* ^−/−^ A-MDSCs *in vivo* may result from the interaction of VLA-1 on A-MDSCs with splenic collagen IV. To address this, we tested motility and migration parameters of WT or *Itga1* ^−/−^ A-MDSCs on collagen IV- or fibronectin-coated glass slides in the presence of Teff (**Figure 4A**). No changes in track speed, track length or track displacement length could be detected between WT or *Itga1* ^−/−^ A-MDSCs and all these parameters were similar on both extracellular matrices (**Figure 4B**). Then we assessed the interactions of M-MDSCs with the T cells added to the assay. In the same experimental settings as before, we found similar interaction times of WT or *Itga1* ^−/−^ A-MDSCs with Teff on fibronectin while interaction times of *Itga1* ^−/−^ A-MDSCs were significantly shorter on collagen IV than of WT A-MDSCs (**Figure 4C**). These data suggest, that collagen IV as a known ligand for VLA-1 does not influence A-MDSC motility and migration, but controls their interaction with Teff by interaction with collagen IV and thereby influence the T cell suppression observed above.

### Intravital 2-photon Microscopy Confirms Deficits of *Itga1−/−* A-MDSCs in Teff Interactions

**Figure 5:**
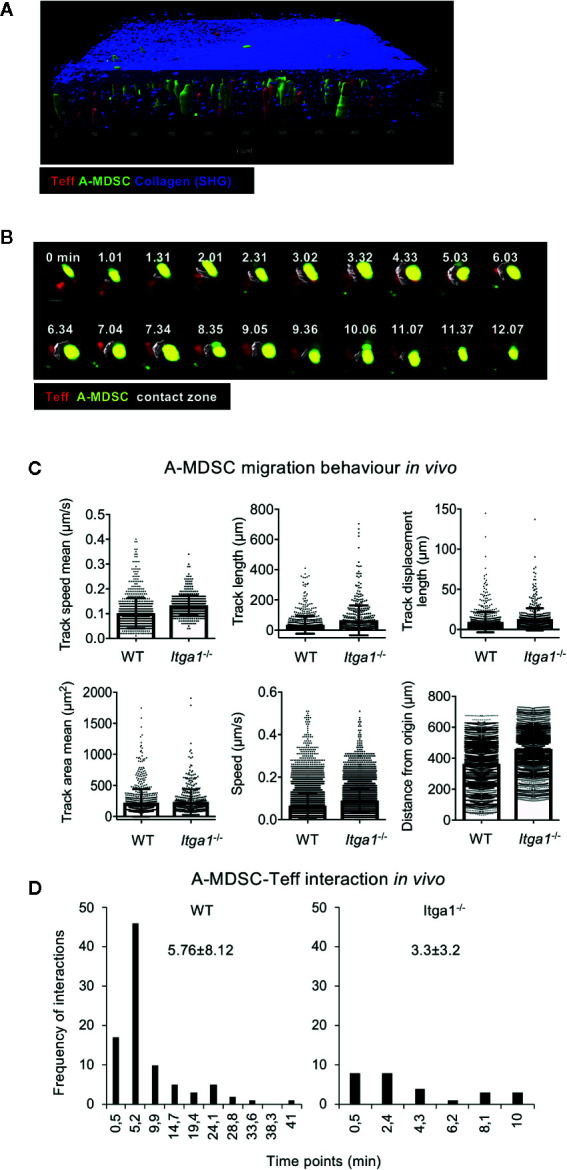
Activated MDSC interaction with Teff in the red pulp requires VLA-1 expression, but is not needed for migration. **(A)** Teff co-localize with A-MDSC in the subcapsular area represented by a thick layer of collagen (blue; SHG, second harmonic generation). CD4^+^ OT-II dsRed Teff were and injected intravenously together with CFSE-labeled A-MDSCs at a 1:1 ratio (7 × 10^6^ cells of each). After 1 h mice were subjected to live two-photon microscopy of the spleen. Representative of n=2 experiments. **(B)** Experimental setup as for **(A)** Representative example of Teff:A-MDSC interaction *in vivo* overtime. A contact zone (white) was created as a surface to highlight the interacting area between the two cell types. The complete sequence of consecutive time points is shown to visualize the duration of the interaction, from its origin until its termination. **(C)** CD4^+^ OT-II dsRed Teff and CFSE-labeled LPS/IFN-γ treated A-MDSCs were injected intravenously into C57Bl/6 mice at a 1:1 ratio. After 1 h mice were subjected to live two-photon microscopy of the spleen. The indicated parameters for cell motility and migration were analyzed by Imaris software. A total of 749 tracks were detected for WT MDSCs and 489 tracks for *Itga1* ^−/−^ MDSCs. **(D)** Experimental set-up as in **(A)** Interactions of WT and *Itga1* ^−/−^ MDSCs were quantified. From the total amount of tracks, 91 (12.14%) resulted in WT MDSC-T cells interacting tracks with an interaction mean value of 5.76 min and 27 (5.52%) interactions of *Itga1* ^−/−^ MDSC-T cells with a mean value of 3.2 min.

To test whether such differences could also appear in the spleen *in vivo*, we followed MDSC-T cell interactions by two-photon microscopy in living mice. Adoptively transferred CFSE-labeled A-MDSCs and dsRed-expressing OT-II Teff cells were observed to localize together in the subcapsular red pulp area of the spleen starting at 1 h after i.v. injection (**Figures 5A, B**). These data also demonstrate the technical feasibility of this approach despite the high density of erythrocytes. Therefore, and for the mentioned technical reasons of light absorbance by erythrocytes, imaging acquisition was particularly focused on the subcapsular region of the red pulp with a range of 70–90 μm in the z-plane. When motility and migration parameters such as track speed mean, track length, track displacement length, track area mean, speed or distance from the origin were recorded, again, no difference was observed between WT and *Itga1* ^−/−^ A-MDSCs (**Figure 5C**). Similar to the *in vitro* contacts with Teff cells, also here WT A-MDSCs showed prolonged interaction times with Teff as compared with *Itga1* ^−/−^ A-MDSCs (**Figure 5D**). These data corroborate the *in vitro* findings that VLA-1 expression on A-MDSC is not required for homing, motility and migration but as a substrate to efficiently interact with activated T cells.

## Discussion

VLA-1 is widely expressed on mesenchymal and immune cell types and less on epithelial cells. It is known to transmit anti-apoptotic, proliferative and tissue retention signals to T cells but also to monocytes after binding to collagen (14). Here we report the expression of VLA-1 on M-MDSCs and addressed its functional role for MDSC spleen homing, motility and migration and its role in the interaction with T cells to mediate suppression. The results indicate that VLA-1 does not serve as a homing receptor into the spleen or for motility or migration within the spleen, but by binding to collagen IV controls the interaction time with VLA-1^+^ Teff. As a consequence, MDSC deficient for VLA-1 show reduced suppression as measured for T cell proliferation and cell death.

We found by bioluminescence imaging and FACS analyses that many of our injected MDSCs reach the lung of healthy mice, however the second highest accumulation occurred in the spleen, but hardly any cells reached the lymph nodes or BM. These data support the idea that a primary function of MDSCs is not to prevent T cell priming but to control Teff activity during chronic stages of diseases in the target organ and systemically when circulating through the splenic red pulp (20, 22). The splenic subcapsular red pulp represents a reservoir for monocytes that are mobilized as an emergency recruitment to inflammatory sites (8). It has been observed that myeloid cells from the spleen can infiltrate tumor stroma to mediate suppressive functions on T cells (36, 37). The accumulation of MDSCs in the spleen has also been reported during trauma and sepsis and, remarkably, also in this report the infiltration occurred already 6 h after stress induction (38, 39). This rapid effect is similar to our 6 h time period required not only to detect A-MDSC and Teff in the spleen but also to show a suppressive and killing effects on T cells. However, we did not find a homing defect of R- or A-MDSCs into the spleen in the absence of VLA-1 expression.

VLA-1 expression has been reported for monocytes before. Activation signals as the combination of LPS + IFN-γ have been described to induce their VLA-1 expression (40). Here, we found that GM-CSF culture increased VLA-1 expression on monocytes but not their further activation by LPS + IFN-γ. This may indicate that both treatments can up-regulate VLA-1 on monocytes but in combination without an additional effect. In contrast, LPS + IFN-γ signals of GM-CSF-treated monocytes induced their release of NO to suppress T cell proliferation, which was not observed without pre-treatment with GM-CSF (5, 7). We described the GM-CSF treatment of monocytes before as a “licensing” process for monocytes converting them into R-MDSC, a prerequisite for their further activation into A-MDSC (7). Our data here indicate that elevated VLA-1 expression serves as another monocyte licensing marker.

Inflammatory stimuli induced VLA-1 expression on the monocytic THP-1 cell line (41), and on activated monocytes in murine TNBS- or DSS-induced colitis models (42, 43). However, the inflammatory circumstances of bypass surgery in monocytes up-regulated only VLA-2 but not VLA-1 (44). Functionally, VLA-1 may support the recruitment of monocytes to inflammatory sites such as by collagen XIII into fibrotic lesions (45) and after monocyte differentiation to macrophages their VLA-1 expression may block their exit from inflamed tissues by collagen IV signals (46). The macrophage migration on collagen IV in chemotaxis assays was markedly inhibited by VLA-1-deficient macrophages (46), suggesting that VLA-1 may serve myeloid cells as a homing receptor and coordinator of migration within tissues (47). Here we show that motility and migration of A-MDSCs were not impaired on the low affinity binding substrate fibronectin or the high affinity binding substrate collagen IV *in vitro* and *in vivo* within the splenic red pulp, an area known to expose collagen IV (15, 16). In contrast, our data indicate that A-MDSCs require VLA-1 interference with collagen IV to prolong the interaction times with VLA-1^+^ Teff contributing to optimal T cell suppression.

VLA-1 expression on T cells has been described mainly for pathological situations such as the murine lung where it was increased after viral infection (21, 48) or on Teff in the psoriatic epidermis of mice (49), murine colitis (42) or human atherosclerotic plaques (50). Among T cells, IFN-γ producing CD4^+^ Th1 cells appear to express the highest levels of VLA-1 in mice and humans (29, 48). Thus, VLA-1 marks Th1 cells homing to inflamed or infected peripheral tissues. Despite this peripheral organ-specific homing under inflammatory conditions, VLA-1^+^ CD4^+^ Teff are also detectable in the spleen (51). Our data concur with this observation since our intravenously injected Teff stay at least for 6 to 24 h in the splenic red pulp and can be targets of suppression when encountering A-MDSCs.

Inhibition of T cell proliferation and induction of cell death are hallmarks of MDSC suppression (52). Here, we generated Teff by antigenic stimulation of OT-II cells for at least 6 days *in vitro*. After adoptive transfer they continue to express Ki-67, as an indicator of ongoing proliferation. Since no further antigen was provided to the OT-II cells after injection *in vivo*, proliferation was only transient. Teff suppression of proliferation and induction of apoptosis was detected 6 h after injection of WT A-MDSCs. This immediate suppressive effect and a trend for apoptosis was partially dependent on the VLA-1 expression by the A-MDSCs, which correlated with the observed general loss of T cells in the spleen. Also, the effector markers CD44 and as a trend CD62L were reduced, potentially also as indicators of cell death. Surprisingly, the expression of the activation marker CD69 was generally higher with A-MDSC co-injection, while CD25 remained unaffected. This may indicate that CD69 expressing T cells are protected from A-MDSC killing to some extent. Injection of *Itga1* ^−/−^ A-MDSCs restored Teff proliferation but did not significantly revert their apoptosis or cell recovery, as compared to WT A-MDSC injection. Notably, CD69 is also considered as a marker of resting tissue-resident T cells (53), it is tempting to speculate if some of our *in vitro* activated and transferred OT-II Teff may have acquired a tissue-resident phenotype in the spleen and are then partially protected from apoptosis induction. Since we found differences in the susceptibility of T cell suppression *in vitro* also here, it will be interesting for future work to investigate the susceptibility of MDSC suppression of different T cell subsets and effector stages in more detail *in vivo*.

MDSCs use multiple mechanisms for suppression, many of them identified from tumor-induced MDSCs (2). Prominent mechanisms include Agr-1- and IDO-mediated metabolic starvation of T cells by preventing their arginine and tryptophan consumption, respectively (54). LPS/IFN-γ stimulation of the granulocytic MDSC subset in our cultures, did not activate their suppressor function. These cells require other stimuli such as zymosan to up-regulate Arg-1 expression (unpublished results). Previously, we found that up-regulation of IDO for tryptophan degradation was induced after LPS/IFN-γ treatment in human CD14^+^ HLA-DR^low^ monocytic MDSCs, whereas the same stimuli and signaling pathways induced iNOS and NO release in their murine Ly-6C^high^ monocytic MDSC counterparts (7). In this study, we did not separately asses the *in vivo* mechanism of suppression employed by the *in vitro* generated and injected MDSCs. However, our previous work and data presented here showed that LPS plus IFN-γ stimulation of monocytic cells in our day 3 GM-CSF cultures (monocytic R-MDSC) led to iNOS induction and NO release to suppress proliferation of T cells or induce T cell and/or DC killing *in vitro* and *in vivo.* Blocking iNOS pharmacologically or using MDSC generated from *NOS2* ^−/−^ mice strongly inhibited suppression, indicating that this is their major suppression mechanism (5, 6, 9). Thus, the mechanism of Teff suppression by LPS/IFN-γ treated M-MDSCs in the splenic red pulp, detected in this study, is strongly suggested to follow the same signaling pathway and mechanism.

The experiments performed with two-photon microscopy allowed the observation of MDSC migration and interaction with T cells only 60–90 µm deep below the spleen capsule due to the high density of erythrocytes that were absorbing light. Thus, the data acquired represent mostly subcapsular red pulp areas. Therefore, our data may not allow extrapolation of our conclusions on deeper red pulp areas. However, the subcapsular areas are known reservoirs of monocytes (8). Our data suggest that the red pulp may also represent a major site of T cell suppression by MDSCs due to specific accumulation of injected A-MDSCs in the subcapsular areas.

In conclusion, we found that high VLA-1 expression is a major characteristic of GM-CSF-licensed monocytes, which represent resting M-MDSCs that can be activated to exert suppressor function on T cells. Teff are more susceptible to MDSC suppression *in vitro* and are specifically and very rapidly targeted by adoptively transferred A-MDSCs in the splenic subcapsular red pulp. Although VLA-1 was mainly reported to serve as a homing receptor for T cells and monocytic cells, we found that the expression of VLA-1 on M-MDSCs was not required to mediate their motility or migration on fibronectin or collagen IV *in vitro* or their homing into the spleen. Instead, our data indicate that VLA-1-mediated contact of A-MDSC to collagen IV is required to prolong the interaction time period with Teff needed to optimize the suppressive effect. Together, we introduce VLA-1 as a novel marker for M-MDSCs with functional relevance for their T cell suppressor function in the spleen.

## Data Availability Statement

The raw data supporting the conclusions of this article will be made available by the authors, without undue reservation.

## Ethics Statement

The animal study was reviewed and approved by Regierung von Unterfranken, AZ 55.2-2532-2-200.

## Author Contributions

IE, ER, SS, SP, and KJ performed the experiments and analyzed the data. IE, ER AB, and ML analyzed and evaluated the data. IE, ER, and ML wrote the paper and prepared the figures. All authors contributed to the article and approved the submitted version.

## Funding

This project was supported by the DFG (LU851/6-2) and the University of Würzburg in the funding programme Open Access Publishing.

## Conflict of Interest

The authors declare that the research was conducted in the absence of any commercial or financial relationships that could be construed as a potential conflict of interest.
